# Long-Term Cryostorage of Mesenchymal Stem Cell-Containing Hybrid Hydrogel Scaffolds Based on Fibrin and Collagen

**DOI:** 10.3390/gels6040044

**Published:** 2020-11-25

**Authors:** Marfa N. Egorikhina, Yulia P. Rubtsova, Diana Ya. Aleynik

**Affiliations:** Federal State Budgetary Educational Institution of Higher Education, Privolzhsky Research Medical University of the Ministry of Health of the Russian Federation (FSBEI HE PRMU MOH), 603600 Nizhny Novgorod, Russia; rubincherry@yandex.ru (Y.P.R.); daleynik@yandex.ru (D.Y.A.)

**Keywords:** scaffold, collagen, fibrinogen, biopolymers, mesenchymal stem cells, cryoprotectors

## Abstract

The most difficult issue when using tissue engineering products is enabling the ability to store them without losing their restorative capacity. The numbers and viability of mesenchymal stem cells encapsulated in a hydrogel scaffold after cryostorage at −80 °C (by using, individually, two kinds of cryoprotectors—Bambanker and 10% DMSO (Dimethyl sulfoxide) solution) for 3, 6, 9, and 12 months were determined, with subsequent assessment of cell proliferation after 96 h. The analysis of the cellular component was performed using fluorescence microscopy and the two fluorochromes—Hoechst 3334 and NucGreenTM Dead 488. The experimental protocol ensured the preservation of cells in the scaffold structure, retaining both high viability and proliferative activity during storage for 3 months. Longer storage of scaffolds led to their significant changes. Therefore, after 6 months, the proliferative activity of cells decreased. Cryostorage of scaffolds for 9 months led to a decrease in cells’ viability and proliferative activity. As a result of cryostorage of scaffolds for 12 months, a decrease in viability and proliferative activity of cells was observed, as well as pronounced changes in the structure of the hydrogel. The described scaffold cryostorage protocol could become the basis for the development of storage protocols for such tissue engineering products, and for helping to extend the possibilities of their clinical use while accelerating their commercialization.

## 1. Introduction

The high demand for tissue engineering products is leading to searches for new solutions to meet clinical needs for tissue replacement materials. One of the most rapidly advancing areas of regenerative medicine is the active development of complex constructs based on scaffolds of various compositions and structures containing cultured cells, and intended to replace damaged and/or lost tissues and organs. Examples of such constructs are the biological equivalents of skin, formed from various matrices and types of cultured cells (keratinocytes and/or fibroblasts). Some of them, for example, Apligraf^®^ (Organogenesis, Canton, Ohio, USA) and Dermograft^®^ (Advanced Biohalin, Inc. La Jolla, California, USA), have reached the stage of commercial realization and have been in clinical use for many years [1,2,3,4]. The demand for bioengineering products is constantly growing, providing the incentive to search for new solutions for both scaffold carriers and sources of appropriate cellular material. Currently, the most attractive option for providing the cellular component is the use of mesenchymal stem cells (MSCs)—cell populations with low immunogenicity and high proliferative activity [5,6]. The easy-availability of the source material is of great importance for practical use. Products in which bone marrow/adipose tissue-derived/umbilical cord/placental MSCs, etc. are used as the cellular component have been developed [7,8,9]. Adipose tissue-derived MSCs are considered to be some of the most readily available cellular materials. It has been proven that this cell population meets all the criteria defined for MSCs by the International Society for Cellular Therapy [10,11,12]. The use of adipose tissue as a source for the isolation of MSCs means that it is possible to obtain a large amount of material during standard liposuction surgeries in cosmetic clinics. Since, when cooled to a certain temperature, the biological activity of cells can be suppressed, with the functions of the cells being restored after thawing, cryopreservation of such cells has become a widely accepted method for the long-term storage of living cells. Protocols for the cryostorage of cell products represented by a cell culture without a carrier are well proven [13,14,15]. However, there are only a few papers devoted to the development of cryostorage protocols for tissue engineering products that include both the scaffold carrier and a cellular component [16,17].

One of the promising areas of tissue engineering is the development of hydrogels that can act as cell carriers. Hydrogels are crosslinked networks of hydrophilic polymers and have several advantages over other materials [18]. Therefore, the ability to retain water, their porous structure, soft nature, and their ability to provide transport of oxygen and nutrients mean that hydrogels closely imitate biological tissues, making them suitable for cell placement. Some hydrogels have already been commercialized and are used, for example, as wound healing dressings [19]. However, no products based on hydrogels that also contain a cellular component in their structure are currently present in the market. Commercialization, ideally, assumes generating a certain amount of finished product with the possibility of preserving it after production and delivery to the consumer. In clinical practice, tissue engineering products can be in demand for a short period when a patient is admitted to the hospital or when a doctor decides to use such a product according to vital indications, for example, to close large areas of burn wounds after early necrectomy. The latter assumes the availability of a ready-made product that can be used in the clinical situation, requiring only short-term preparation. Thus, clinical and market demand dictates the need to develop tissue-engineered products that do not lose their restorative capacity during storage.

We recently described an original product based on a hybrid hydrogel scaffold made of biopolymers and human adipose tissue-derived MSCs (ASCs) [20]. The possibility of long-term cryostorage of this product has been assessed to ensure future clinical use.

The goal of this study was to assess the possibility of, and conditions for, the cryopreservation of a hybrid hydrogel scaffold able to provide the characteristics of viability and proliferative activity of its cellular component after long-term cryostorage.

## 2. Results and Discussion

**Figure 1:**
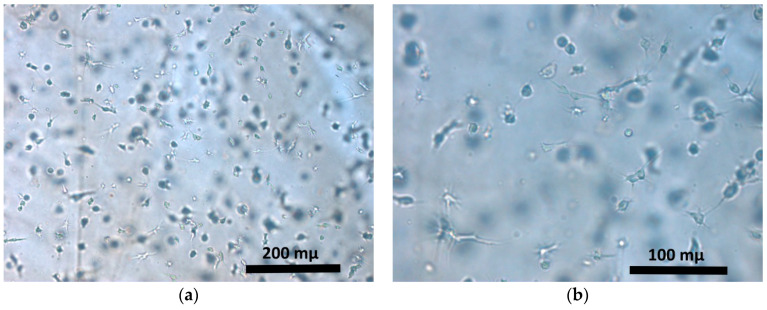
ASCs in the scaffold structure after 72 h of cultivation: (**a**) objective lens 10×, eye-. piece 10×, light microscopy, (**b**) objective lens 20×, eyepiece 10× light microscopy.

Hybrid hydrogel scaffolds were cultured for 72 h under standard conditions, after which the ASCs encapsulated in the scaffolds showed three-dimensional growth, had put out cell processes and formed intercellular contacts (Figure 1). This was the stage at which the scaffolds were frozen.

During cultivation, the total number of cells and the number of dead cells within the structure of the scaffolds in the samples were determined. Before cryopreservation (72 h), the total numbers of cells in all groups (3, 6, 9, 12 months) were not statistically significantly different (Table 1), and none of them had a significant number of dead cells (Table 2). In fact, the number of dead cells in the scaffolds before cryopreservation is best characterized as a single cell or complete absence in each field of view. This number did not exceed 1% of the total number of cells. Upon a further 72 h cultivation of scaffold samples from the same groups, a statistically significant, almost two-fold increase in the total number of cells within the scaffold structure was observed. After 144 h of cultivation, there was a slight increase in the number of dead cells, but it remained at an extremely low level, at most 1%.

After cryopreservation of the scaffolds for 3 months followed by a further 24 h-cultivation, the number of cells per 1 mm^3^ of the scaffolds had increased more than 1.5-fold compared to that before cryopreservation (72 h). This was typical for both types of cryoprotectants (Table 1). When the scaffolds were cultured for 96 h after defrosting, we observed a statistically significant increase in both the total number of cells, and a tendency for the number of dead cells to increase. It should be noted that the number of cells after cryopreservation and 96 h of culturing of the scaffolds was comparable to the number of cells present in the group after 144 h of culture without cryopreservation. The number of dead cells in all groups after three months of conservation was quite small and amounted to no more than 2% of the total number of cells (Table 2). This aspect was typical for both types of cryprotectants.

**Figure 2:**
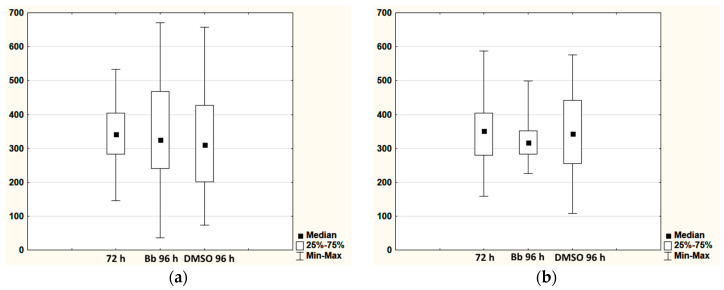
Comparative diagrams for the loaded hydrogels cryostored for (**a**) 6 months, (**b**) 9 months.

A similar picture was observed after 6 months of storage. The total number of cells per 1 mm^3^ of the scaffold cultured for 24 h after defrosting had increased compared to their number before cryopresevation (72 h). However, cultivation of the scaffolds for 96 h after defrosting did not lead to a further increase in the number of cells. The total number of cells was comparable to that before cryopreservation (72 h). However, statistical analysis showed that there were statistically significant differences in the Bb (96 h) and DMSO (96 h) groups compared to the initial concentration (72 h). The revealed statistical differences were, apparently, associated with changes in the distribution of data within the groups, this being well demonstrated by the range diagrams (Figure 2a). The number of dead cells in the scaffolds after cryopreservation did not exceed 1.5% in any of the groups (Table 2).

After 9 and 12 months of cryopreservation and a subsequent 24 h of cultivation, the results from both types of cryoprotectants showed an increase in the total number of cells per unit volume compared to their number before cryopreservation by a factor of 1.5 on average (Table 1). This picture was comparable to the results of 3 and 6 months storage of the scaffolds. After further cultivation (96 h) of the scaffolds that had been stored for 9 months, it was detected that the total number of cells remained comparable to that before cryopreservation (72 h). As with the groups of scaffolds that had been stored for 6 months, differences in the distribution of data within the groups caused statistical differences between the groups Bb (96 h), DMSO (96 h) and the initial concentration (72 h) (Figure 2b). A significant increase in the number of dead cells was found for the Bb and DMSO groups after 9 months of cryopreservation, both in comparison with the data before cryopreservation and the groups that had gone through only 3 or 6 months of cryopreservation (Table 2). The total number of cells per 1 mm^3^ in the scaffolds after 12 months of storage and 96 h of cultivation had not changed compared to that found for this group after just 24 h of cultivation following defrosting. At the same time, the number of dead cells increased sharply in comparison with the groups before freezing and following the shorter storage periods, and ranged between 34% and 48%.

It should be noted that the number of dead cells in scaffolds after cryopreservation for 3 and 9 months and subsequent cultivation (24 h, 96 h) was less than 1.5%. That was characteristic of both the Bb and DMSO groups. The picture of what was happening has changed significantly after 9 and 12 months of cryostorage. The number of dead cells was sharply increasing at all stages of cultivation after cryostorage (24 h, 96 h). There was a trend towards an increase in the number of dead cells in the DMSO groups compared to Bb.

**Figure 3:**
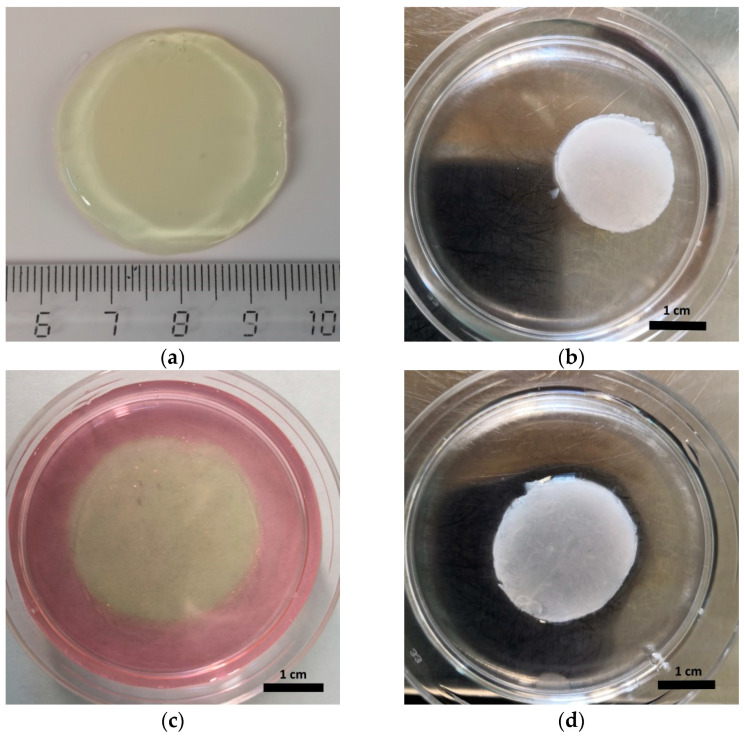
Representative photographs of scaffolds before and after cryopreservation: (**a**) typical appearance of scaffold before cryopreservation; (**b**) appearance of a scaffold after 3 months of cryopreservation and 24 h of culturing (scaffolds look identical after 3, 6, 9 and 12 months of cryopreservation and 24 h of culturing); (**c**) appearance of scaffolds after 3 months of cryostorage followed by 96 h cultivation; (**d**) appearance of scaffolds after twelve months of cryostorage followed by 96 h cultivation.

When conducting a visual assessment of the state of the scaffolds, it was noted that after cryopreservation for all the periods studied, they had changed in both color and transparency (became white and non-transparent) and had decreased in size compared to that before cryopreservation (Figure 3a,b). The change in size could be characterized by the density of the structure. Scaffolds, after cryopreservation for 3, 6, and 9 months and cultured under standard conditions for 96 h, were observed to be recovering their shape and transparency (Figure 3c). However, scaffolds that had been cryopreserved for 12 months did not recover their previous parameters during 96 h of cultivation under standard conditions (Figure 3d).

When analyzing the literature data on the cryostorage of tissue engineering products based on cell-loaded hydrogels, it was noted that, as a rule, only the short-term storage effect on product morphologies/appearence for a period of up to 7 days was estimated [21,22,23]. At the same time, the possibility of cryostorage for up to 10 years has been demonstrated for cell products without a carrier [24,25]. The possibility of long-term storage of cell products significantly expands the prospects for their use and commercialization. On this basis, we have made it our mission to assess the possibility of long-term storage (from three to twelve months) of the hybrid hydrogel scaffolds, containing encapsulated ASCs that we have developed. According to the reported data, based on experimental studies and clinical experience, a sufficient number of living cells is critically important to ensure the regenerative process when using cell products. O.W. Gramlich et al. posited that the predictor of MSC function is the viability of the mesenchymal stem cells, rather than the cryopreservation itself [26]. In this regard, one of the main criteria for assessing the safety of cell products is the assessment of cell viability after thawing [27,28]. We estimated the total number of cells per unit volume of the scaffolds and the percentages of dead cells. When assessing the results, it was found that the total number of cells after cryopreservation at all periods was significantly increased in comparison to the number that was fixed before cryopreservation. Such a significant increase could not be associated with cell proliferation, as 24 h of cultivation after cryopreservation is too short for the actual number of cells to increase by 1.5 times or more. However, when assessing the visual characteristics of the scaffolds after cryopreservation, a significant decrease in their size was noted. A comparison of these data suggests that the observed increase in the total number of cells per 1 mm^3^ of the scaffold is associated with the “shrinkage” of the scaffold structure. The “shrinkage” effect of the scaffold can be associated with the effect of osmotic shrinkage. For the cryopreservation, we applied well-known cryoprotectors with a penetrating effect used for cell cryostorage. The principle of operation of cryoprotectors is based on their action of water replacement, on one hand, and of binding them with water molecules (via hydrogen bonding), on the other hand, to prevent ice formation at the cryopreservation temperature [29]. When replacing the water in a cell with a cryoprotector, the cell first shrinks due to osmosis, and then becomes swollen due to the penetration of the cryoprotectors into the cell. This is a classic reaction known in cryobiology as shrink swell [30]. The reaction proceeds until the volume of the cryoprotectors is equal to that of the original intracellular and extracellular fluids of the cells. The rate at which cryoprotectors enter cells varies for different cell types and cell sources [31,32]. An important role in this is played by the cell membrane and the membranes of the intracellular organelles, these having selective permeability that is involved in regulation of the process. Considering that hydrogels can contain up to 99% water, and the interaction with the cryoprotector is not limited by membranes (as with the cells), it can be assumed that osmotic shrinkage during the interaction of the cryoprotector with the hydrogel is likely to be more pronounced. It is likely that the replacement of a large volume of liquid in the hydrogel by a cryoprotector that is not regulated by any membrane will lead to “shrinkage” of the hydrogel structure. With further cultivation of the scaffolds (96 h) that had undergone 3, 6, and 9 months of cryostorage, the volume of the hydrogel was restored, and the appearance of the scaffolds corresponded to that before cryopreservation. After 3 or 6 months of cryostorage the cells retained high viability. However, in the hydrogel scaffolds stored for 9 and 12 months, a significant increasing in the number of dead cells was recorded. The latter is the basis for concluding that a useful cryostorage of the presented hydrogel scaffolds for 9 or mostly 12 months, using the protocol approved in this work, is not possible. This is because the loss of viability of the cells will inevitably lead to a decrease in the restorative capacity of the product.

When assessing the total number of cells after 96 h of cultivation, it was demonstrated that the number of cells in scaffolds stored for 3 months had increased compared to 24 h of cultivation, in contrast to the scaffolds stored for 6 or 9 months. Considering that the scaffolds cryostored for 3 months of storage had restored their volumes, becoming comparable to that without cryopreservation for which the cell number rose after 144 h of cultivation, it can be concluded that the cells retained their proliferative activity. The total number of cells per 1 mm^3^ in the scaffolds cryopreserved for 6 or 9 months and after 96 h of cultivation is similar to that corresponding to the systems before cryopreservation (72 h) and in a marked contrast to the number of cells in the scaffolds cultivated for 144 h (without cryopreservation). Practically, ASCs in scaffolds cryostored for 6 and 9 months did not recover their proliferative activity. A decrease in cell proliferative activity is a negative result. It is known that the ability to proliferate in vitro is one of the key characteristics of MSCs [33,34]. A decrease in the proliferative activity of MSCs is a critical indicator of an abnormality in functional activity of cells, which can be caused both by natural processes, for example, cell senescence [35,36], and by the damaging effect of various external factors [37,38]. A decrease in the proliferative activity of stem cells may be accompanied by a decrease in their metabolic activity, including after cryostorage [39]. Thus, the dysfunction of the proliferative activity of cells after cryopreservation indicates that, after six months of cryostorage, the investigated scaffolds cannot be recommended for use in regenerative medicine, despite a high viability of the cells.

Summarizing the results obtained, we can assume that the approved protocol for cryostorage of hybrid hydrogel scaffolds with encapsulated ASCs can deliver the useful storage of scaffolds for three months. During this and subsequently, both high cell viability (at a level of more than 95%) and the proliferative activity of the ASCs in the scaffold structure are well preserved. After thawing, a period of so-called acclimatization is required. Thus, recently, B. Antebi et al. demonstrated that a 24-h period of acclimatization after thawing may “reactivate” the thawed cells and restore their weakened functional activity [39]. We propose acclimatizing the scaffolds within 96 h after thawing, by cultivating them under standard conditions. In our opinion, this will make it possible to restore the functional activity of the cells. Furthermore, it was demonstrated that during cultivation for 96 h after cryopreservation, the scaffold regained its shape after the “shrinkage” caused by cryopreservation. We consider this to be an important aspect. The restoration of the shape indicates the preservation of the polymer structure of the hydrogel and its proper re-swelling with water, which should provide appropriate conditions for cell growth.

The study demonstrated that after twelve months of cryostorage the scaffolds did not regain their shape even after 96 h of cultivation. It is possible, indeed, that this is due to critical dysfunctions in the structure-forming biopolymers. The scaffold presented in the paper is formed from components of blood plasma and collagen. It should be noted that the main structure-forming protein of the scaffold is fibrinogen, the concentration of which was 22 times higher than that of the collagen [20]. It is known that cryopreserved blood plasma can be stored for a fairly long period without damage and loss of activity of the main components [40]. However, while the proteins in cryopreserved blood plasma are in their native state, the scaffold is formed by polymerized proteins organized into a three-dimensional structure [20]. Therefore, it would not be correct to extrapolate the conditions and results of the storage of blood plasma to the results of storage of the scaffolds. Cryostorage can likely cause significant changes in the properties of biopolymers organized into a three-dimensional scaffold structure. Nevertheless, the preservation of the structural elements of the hydrogel should contribute to the preservation of the cellular component of the scaffold. Several studies have demonstrated that the use of hydrogel capsules for the cryopreservation of cells makes it possible to minimize the damaging effects associated with cryostorage [41,42,43]. Hydrogel scaffolds generally have a three-dimensional structure and have biomimetic properties comparable to natural tissues [44,45]. There is evidence that tissue can prevent cell damage during cryostorage by maintaining a natural three-dimensional microenvironment [46]. N. Cagol et al. [47] demonstrated the protective properties of an alginate hydrogel scaffold for cells during cryostorage. Cell viability and their functional activity were significantly better in those cells loaded in the scaffold during cryostorage than in cells cryopreserved simply in suspension [47]. Furthermore, the duration of cryopreservation was fairly short (less than two weeks) in all of the above-mentioned studies. The authors did not describe the state of the hydrogel itself after cryostorage but focused only on the characteristics of the cells. We believe that the changes in the state of the hydrogel that we have revealed during long-term cryostorage require further study. Understanding the mechanisms of these changes will allow the development of cryostorage protocols for tissue engineering products based on hydrogel scaffolds in the future, and this should make it possible to preserve such products for a long time without losing their regenerative and restorative capacity.

## 3. Conclusions

The protocol described for the cryostorage of a hybrid hydrogel scaffold with encapsulated ASCs allows the scaffold to be retained for three months while maintaining both high cell viability and proliferative activity. Additional studies are required to confirm the preservation of the metabolic and developmental potential of the cells kept under these conditions, thereby justifying the maintenance of the restorative capacity of the scaffold for future clinical use. We revealed staging of the changes occurring with the scaffold and cells during storage over periods exceeding three months (6 months—a decrease in cell proliferative activity, 9 months—a decrease in both the viability and proliferative activity of the cells, 12 months—changes in the structure of the hydrogel and a decrease in both the viability and proliferative activity of cells), suggesting the value of developing protocols for longer cryostorage that will slow down or eliminate the observed changes during these periods. The presented protocol for scaffold cryostorage can therefore become the basis for the development of new storage protocols for such tissue engineering products, which will help to extend their clinical use and accelerate their commercialization.

## 4. Materials and Methods

### 4.1. Compliance with Legal Regulations and Ethical Norms

All donors of biological material provided informed consent and voluntarily approved the collection and use of their biological material. All donors were examined by specialists to establish that none had vector-borne infections.

The research protocol was approved by the local ethics committee of the Federal State Budgetary Educational Institution of Higher Education, the “Privolzhsky Research Medical University” of the Ministry of Health of the Russian Federation (FSBEI HE PRMU MOH) and acknowledged by the Academic Council, the FSBEI HE PRMU MOH.

### 4.2. Hybrid Hydrogel Scaffolds

The scaffolds were formed under conditions of enzymatic hydrolysis with the use of human blood plasma cryoprecipitate and type I collagen isolated from cod skins (2% solution; pH = 7.4) [20,48]. The blood plasma cryoprecipitate was preliminarily PEGylated (PEG-NHS, Sigma-Aldrich, Darmstadt, Germany). During the formation of the scaffolds, ASCs were introduced into the composite (concentration of cells per 1 mL of the composite was 1.2 × 10^5^). The composite was injected with a thrombin–calcium mixture: 80 IU/mL of human thrombin (NPO RENAM, Russia) in 1% CaCl_2_ solution. Scaffolds were formed within 20 min at a temperature of 22–25 °C.

The plasma cryoprecipitate was obtained using a method previously described by ourselves [20] with a subsequent standardization of the fibrinogen concentration (6 g/L). Frozen human blood plasma (−40 °C) was obtained from the Nizhny Novgorod Blood Center. The scaffolds were flooded in complete growth medium (α-MEM medium, 20% fetal calf serum (FCS), antibiotics and glutamine) and cultured under standard conditions in a CO_2_ incubator (37 °C, with a humidified atmosphere and 5% CO_2_ content).

Manipulations with blood plasma and cryoprecipitate regarding the isolation and culturing of cells, as well as the formation and cultivation of the hydrogel scaffolds, were carried out under class A laminar sterile conditions in the FSBEI HE PRMU MOH biotechnology laboratory. During the cultivation of cells and hydrogel scaffolds, the growth media were regularly monitored for sterility, checking for contamination with mycoplasmas, viruses and the presence of fungal flora.

### 4.3. Cell Cultures

To obtain a culture of ASCs, human adipose tissue was used. This was obtained while carrying out cosmetic surgery operations at the Federal State Budgetary Educational Institution of Higher Education, the “Privolzhsky Research Medical University” of the Ministry of Health of the Russian Federation (FSBEI HE PRMU MOH). Isolation of cells was achieved by thermal enzymatic treatment (1 h, 37 °C, collagenase enzyme (Sigma-Aldrich, Darmstadt, Germany)). The ASCs were cultured in complete growth medium under standard conditions. For the formation of the scaffolds, cultures of the third passage were used. The cells used for the formation of the scaffolds corresponded to criteria defined for mesenchymal cells by the International Society for Cellular Therapy [12]. The cells expressed CD105 +, CD90 +, CD 44+ and CD 73+ and did not express CD 14-, CD 45- and HLA DR- (monoclonal antibodies CD 105 PE, CD 90 FITC, CD 44 FITC, CD 73 PE, CD 14 PC5, CD 45 PC5 and HLA-DR PC7 Becton Dickinson, Franklin Lakes, New Jersey, USA) with the corresponding isotypic controls: flow cytofluorimeter BD FACS CANTO II (Becton Dickinson, Franklin Lakes, New Jersey, USA). The cells spread well on a plastic surface and were capable of differentiating in three directions: adipogenic, osteogenic and chondrogenic (Hyman Mesenchymal Stem Cell Functional Identification Kit (R and D systems, Minneapolis, MN, USA)). The viability of the cells introduced into the scaffold composition was 98–99%.

### 4.4. Cryostorage of Scaffolds

Once formed (Section 4.2), the scaffolds were cultivated under standard conditions in complete growth medium for 3 days. Then each was divided into 4 parts. On the first part, a quantitative analysis of cells was performed (Section 4.6). The second part was cultivated for a further 3 days (making the total cultivation period 6 days), after which a quantitative analysis of its cells was carried out (Section 4.6). The third and fourth parts were exposed to cryopreservation. For this, the samples were washed three times in a phosphate buffer solution to remove the culture medium. Then they were placed in cryovials (2 mL, Corning) containing 1.5 mL of cryopreservative: for the third part, we used Bambanker^TM^ (further Bb) cell freezing medium (Lymphotec, Tokyo, Japan), while the fourth part was placed in a 10% DMSO solution (Sigma-Aldrich, Darmstadt, Germany). The cryovials with the scaffold samples in the cryoprotectants were kept at room temperature for 30 min before freezing. The cryotubes, housed in specialized containers for freezing, were stored in a refrigerator (SANYO Electric Co., Moriguchi, Osaka, Japan) where the temperature was maintained at −80 °C. At the targeted timeframe, after 3, 6, 9, 12 months of cryostorage, the samples were examined. For this, the cryovials with samples were removed from the refrigerator and placed in a thermostat, with the temperature set to 37 °C, for 30 min. Then, the defrosted scaffolds samples were removed from the test tubes, washed three times in phosphate buffer, divided into 2 fragments, and these placed in a 24-well plate. The samples were then cultured in complete growth medium under standard conditions. Twenty-four h after defrosting, a quantitative analysis of the cells was carried out on the first part of each sample (Section 4.6) while culturing of the second part was continued for a total of 96 h after defrosting, after which time a corresponding quantitative analysis of its cells was carried out.

### 4.5. Microscopy

Microscopy (bright field and phase contrast method) and video archiving were performed using a “Leica DMI 3000B” inverted microscope with LAZ software. V. 3.4 (Leica Microsystems, Wetzlar, Germany). Fluorescence microscopy using the Z-stack function was performed on a Cytation 5 imager with Gen 5 Image + (BioTek, Winooski, VE, USA) software.

### 4.6. Quantitative Analysis of Cells in Scaffolds

The analysis of the number of cells was carried out using a method of quantitative analysis of the cellular component of the scaffold [20,49]. The number of cells was determined by counting the vitally stained nuclei of cells in the test sample. For this, the cell nuclei were stained with Hoechst 3334 fluorochrome (catalog No. 561908, BD, Franklin Lakes, NJ, USA)—excitation wavelength of 377 nm and emission wavelength of 477 nm. We analyzed 10 micrographs from each sample taken from several fields of view at arbitrary regions within the thickness of the samples (magnification: 4× objective, 10× eyepiece). The objects were registered in areas of 530 µm along the Z axis. To count the dead cells, the samples had been additionally stained with NucGreenTM Dead 488 fluorochrome (catalog No. R37109, Invitrogen by Thermo Fisher Scientific, Waltham, MA, USA)—excitation wavelength of 477 nm and emission wavelength of 525 nm. A total of 5 micrographs from three samples of each group were analyzed, taken from several fields of view at arbitrary regions within the thickness of the samples (magnification: 10× objective, 10× eyepiece). The objects were registered in areas of 300 µm along the Z axis. Staining had been performed in accordance with the manufacturer’s protocol. Fluorescence microscopy using the Z-stack function was performed on a Cytation 5 imager with Gen 5 Image + software (BioTek, Winooski, VE, USA). Quantitative analysis was carried out using the crosslinked Z-stack micrographs along with counting the number of cell nuclei and a further re-counting of the number of cells per 1 mm^3^.

### 4.7. Statistical Analysis

The research results were processed by nonparametric statistics methods, using Wilcoxon’s paired comparison test with the STATISTICA 6.0 software package.

## Figures and Tables

**Table 1:** The number of cells in scaffolds before and after different cryostage periods.

The Timing of the Cryostorage	Before Freezing (Total Number of Cell Nuclei per 1 mm^3^ Scaffold)		After Freezing (Total Number of Cell Nuclei per 1 mm^3^ Scaffold)			
	72 h	144 h	Bb 24 h	Bb 96 h	DMSO 24 h	DMSO 96 h
3 months	354.13 ± 18.16	616.53 ± 20.71 ○○	548.95 ± 19.21 ○	687.18 ± 19.48 ▪ ○ ●	589.46 ± 26.98 ○○	636.07 ± 17.6 ◊ ○○
6 months	338.16 ± 11.20	640.90 ± 15.40 ○○	513.26 ± 25.15 ○○	339.54 ± 18.48 ▪ ○ ●	510.10 ± 21.89 ○	312.17 ± 18.29 ◊◊◊ ○○ ●
9 months	346.35 ± 12.03	787.91 ± 29.71 ○○	517.70 ± 10.76 ○○	327.17 ± 8.04 ▪ ○	428.76 ± 10.86 ○○ ∆	354.64 ± 16.11 ◊◊ ○ ●
12 months	369.46 ± 21.40	640.90 ± 14.85 ○○	587.83 ± 36.97 ○	592.82 ± 41.63	540.02 ± 21.51 ○	539.42 ± 21.78 ○

Note: ○—*p* ˂ 0.05; ○○—*p* ˂ 0.001—comparison with the 72 h group before cryopreservation; ▪— *p* ˂ 0.001—comparison of groups Bb 24 h and Bb 96 h after cryostorage; ◊—*p* ˂ 0.05; ◊◊— *p* ˂ 0.01; ◊◊◊—*p* ˂ 0.001—comparison of groups DMSO 24h and DMSO 96 h after cryostorage; ●—*p* ˂ 0.001—comparison of groups Bb 96 h and DMSO 96 h with the group from 144 h before cryopreservation; ∆—*p* ˂ 0.05—comparison of groups Bb 24 h and DMSO 24 h after cryostorage.

**Table 2:** The number of dead cells in scaffolds before and after different cryostage periods.

The Timing of the Cryostorage	Before Freezing (Number of Nuclei of Dead Cells per 1 mm^3^ Scaffold)				After Freezing (Number of Nuclei of Dead Cells per 1 mm^3^ Scaffold)							
	72 h	%	144 h	%	Bb 24 h	%	Bb 96 h	%	DMSO 24 h	%	DMSO 96 h	%
3 months	2.48 ± 0.64	0.70	2.64 ± 0.68	0.43	4.89 ± 0.94	0.89	7.99 ± 2.37 ○	1.16	4.58 ± 1.44	0.78	10.32 ± 2.09 ○ ●	1.62
6 months	1.24 ± 0.32	0.37	4.04 ± 1.04 ○	0.63	2.43 ± 1.70	0.47	4.38 ± 2.34	1.29	1.95 ± 0.86	0.38	3.89 ± 2.12	1.25
9 months	2.56± 0.66	0.74	7.06 ± 1.82	0.90	43.59 ± 3.07 ○○	8.42	47.83 ± 7.44 ○○	14.62	50.30 ± 7.04 ○○ ●	11.73	51.79 ± 9.94 ○○ ●	14.60
12 months	1.71 ± 0.44	0.46	3.82 ± 0.98 ○	0.59	205.35 ± 23.24 ○○	34.93	235.52 ± 27.75 ○○	39.73	241.36 ± 37.74 ○○ ●●	44.69	264.2 ± 28.94 ○○ ●●	48.99

Note: %—% of dead cells, relative to the total number; ○—*p* ˂ 0.05; ○○—*p* ˂ 0.001 —comparison with the 72 h group before cryopreservation; ●—*p* ˂ 0.01; ●●—*p* ˂ 0.001; —comparison of groups Bb 96 h and DMSO 96 h with the group from 144 h before cryopreservation.
